# Editorial: Exploring the Potential of Natural Products Through Advanced Techniques and Green Solvents

**DOI:** 10.3389/fchem.2020.627111

**Published:** 2020-12-07

**Authors:** Gerardo Fernández Barbero, Ana Carolina de Aguiar, Marta Ferreiro-González, Mauricio Ariel Rostagno

**Affiliations:** ^1^Department of Analytical Chemistry, Faculty of Sciences, University of Cadiz, Cadiz, Spain; ^2^Laboratório de Engenharia de Processos, Departamento de Engenharia de Alimentos, Faculdade de Engenharia de Alimentos (LEP/DEA/FEA), University of Campinas, Campinas, Brazil; ^3^Department of Analytical Chemistry, Faculty of Sciences, University of Cadiz, Cadiz, Spain

**Keywords:** natural products, green solvents, new technologies, extraction techniques, purification techniques

This Research Topic presents a brief overview on a number of relevant studies that focus on two main aspects, i.e., on the one hand, the potential of natural products of interest obtained through extraction, separation, purification, or transformation by applying novel and advanced extraction and conservation techniques and on the other hand, the study of green methodologies that intend to either minimize the use of harmful solvents or favor the use of green solvents.

A wide and complex range of plants, animals, insects, and microorganisms that can be found in nature exhibit the capacity to produce a broad range of chemical compounds with interesting properties regarding human health and are still to be explored from a diversity of approaches. These include the attempts to replace synthetic food additives for health concern reasons or the production of second generation ethanol from biomass, just to mention a couple of them. In this regards, Science is increasingly discovering the potential of natural products and gradually changing our world and reshaping the way our society contemplates them.

If the use of natural products is to become a reality, there are several aspects which must be taken into consideration. Thus, their effectiveness or even their possible negative effects on human health as well as other aspects such as the methodologies employed in their production, their processing costs and also their environmental impact are important matters of concern. At the same time, the varying form in which they can be found in nature and the specific requirements that to ensure their production according to a satisfactory standard quality so that they are generally accepted by consumers are some of the issues to be seriously considered.

Consequently, natural products are constantly gaining weight and leading to the emergence of new technologies, processes, and methodologies applied to their extraction, separation, purification, transformation or analysis.

Even though considerable advances are being made, the challenge to develop new techniques and more efficient, greener, more rapid and affordable methods is still a pressing need. The use of green solvents, such as CO_2_, water or biobased ones that intend to reduce environmental impact, while improving selectivity and yields, have proven to be of special interest, since they can largely contribute to perfect old production processes, particularly when combined with particularly advanced and innovative techniques. In this sense, supercritical fluid technology deserves special attention for its potential to do away with the use of the toxic or polluting solvents that have been traditionally used by other methods. The extraction of hydrophobic compounds using supercritical CO_2_ or the hydrolysis of cellulose from biomass using subcritical water are only some of the examples of a great potential still to be further investigated. Ultrasound is another technology that has been widely used to enhance some old processes by combining it with a number of new technologies and methodologies. Microwaves and electric fields have also proven their efficiency as an alternative processing method with a great potential.

In order to truly and deeply understand the mechanisms involved, we should still gather a considerable amount of data that would allow us to determine the influence of the process parameters to be considered in relation to each technology; i.e., temperature, pressure, power input, processing time, raw material features, stability, etc. And such knowledge should lead us to a further and more successful development of those attractive and novel technologies.

“Exploring the Potential of Natural Products through Advanced Techniques and Green Solvents” is a special issue of *Frontiers in Chemistry* that intends to illustrate the state of the art in this field through six outstanding contributions.

Chaves et al. contribution consists on an exhaustive revision on the use of modern techniques for the extraction of flavonoids from natural sources. Flavonoids are natural phenolic compounds that are synthesized by some plants as secondary metabolites and can be widely found in the vegetable kingdom. They are responsible for the flavor, color, and pharmacological activities of plants and plant products. Flavonoids exhibit rather marked and interesting biological properties, such as antioxidant, anti-inflammatory or anti-tumor activities, among others. Those beneficial properties make of them the focus of interest for a large amount of projects and papers that are currently addressing a number of specific aspects, such as their biological synthesis, extraction, analysis or stability. In this review, the newest natural compound extraction techniques that are currently being used are addressed in detail. Those techniques intend to minimize solvent consumption, increase extraction yields, improve extraction selectivity, reduce processing times, and minimize the degradation of the target compounds, thus reducing process costs and environmental consequences. ultrasound assisted extraction (UAE), supercritical fluid extraction (SFE), microwave assisted extraction (MAE), pressurized liquid extraction (PLE or accelerated solvent extraction-ASE), along with a combination of different techniques are among those novel techniques that are being employed to extract both flavonoids as well as bioactive compounds from natural sources (Barba et al., 2016).

In another study, Kalra et al. investigated fungi as a potential source of pigments. A growing concern related to the harmful effects of synthetic colorants on both human health and the environment has given place to a substantial interest in natural coloring alternatives. Thus, the world demand for natural colorants that can be used by the food, cosmetic and textile industries is rapidly increasing. At present, plants and microorganisms are the main source of dyes and pigments used by modern industries. Among other unconventional sources of dyes and pigments production, filamentous fungi and, particularly, ascomycete and basidiomycete (mushroom) fungi, and lichens, are known to produce an extraordinary range of colors that comprise several chemical classes of pigments such as melanins, azaphilones, flavins, phenazines, and quinines. Filamentous fungi are an important source of a wide range of metabolites such as polyphenols, polyketides, carotenoids, terpenoids and a broad range of colored compounds. Selecting the extraction techniques is one of the crucial steps for the efficient recovery of these metabolites and it mainly depends on the nature of the metabolites of interest as well as on the location of such metabolites in the mushroom culture (Chadni et al., 2017). Some of the pigments are extracellular and in those cases, they are easily released into the fermentation broth, which makes their extraction generally more feasible than in the case of intracellular pigments, which require specialized extraction techniques to be removed from the biomass containing them. The use of inexpensive, efficient, and safe extraction methodologies for the recovery of natural pigments is one of the main challenges to be overcome in order to enable large-scale production. For easier and more feasible further processing, extracellular, and water-soluble pigments obtained by submerged cultures are preferred. For the extraction of hydrophobic and intracellular compounds, green extraction methods seem to be the most suitable, since they are free from organic solvents or require smaller amounts of the same and are, therefore, considered both safer and more environmentally friendly. Some of these extraction techniques can also be successfully used at low temperature. This contributes to the extraction of thermolabile pigments while avoiding their degradation. UAE, PLE, MAE, SFE, ionic liquid-assisted extraction and pulsed electric field (PEF) assisted extraction are among the latest techniques used to obtain fungal dyes.

With respect to original research articles, Song et al. extracted and synthesized five pyrethroid insecticides by applying, for the first time, deep eutectic solvent micro-functionalized graphene (DES-G) as the adsorbent of the dispersive micro solid-phase extraction (DMSPE). While food safety is generally a matter of major concern for most consumers, pesticides, and other additive residues have always been considered a serious problem when it comes to human safety. The use of certain pesticides such as organochlorine, organophosphorus or pyrethroid insecticides greatly contribute to the control of pests and to the increment of crop yields. However, airborne pesticides may accumulate on the surface of crops and enter the food chain causing considerable damage to consuming species. Pyrethroid insecticides are particularly toxic to mammals because of their low water solubility, high liposolubility, and a considerable capacity to pass through biofilms (Feo et al., 2010). It is therefore necessary to develop the adequate analytical methods that allow for the extraction and analysis of pyrethroid insecticides, especially from complex matrices. In this sense, in order to improve the detection efficiency of pyrethroid insecticide analytical methods and to reduce organic solvent consumption, a green and efficient microextraction method was to be developed. Thus, such a method has been developed in this research, where several DES-Gs have been synthesized by simple procedures and an optimum DES-G has been selected as the adsorbent of DMSPE. The developed method has been successfully applied to determine pyrethroid insecticides residues in beebread, *Curcuma wenyujin* and *Dendrobium officinale* with recoveries in the range of 80.9–114.1%. Another article by Chang et al. described the production of the hydrogen-donor solvent tetralin by means of the direct liquefaction of coal. Pure tetralin liquid as well as mixture of tetralin and Wucaiwan coal (WCW) were separately reacted under a liquefaction conditions. Direct liquefaction of coal is an efficient option for the green use of coal, since it transforms solid coal into liquid solvents by adding hydrogen (Vasireddy et al., 2011). When tetralin is used as the hydrogen-donor solvent the liquefaction rate of WCW at 380 and 420°C reaches between 69.76 and 83.86%, respectively. Xu et al. have studied the extraction and purification of phenylethanoid glycosides (PhGs) from *Cistanche tubulosa*, by adsorption of its extracts. PhGs are the main active compounds in *Cistanche tubulosa*, which exhibit several pharmacological activities including the protection of neurons and liver, the regulation of the immunological system as well as anti-inflammatory and antioxidative properties. For the extraction and purification of these compounds a new adsorption material was tested, namely the mesoporous carbon (Qin et al., 2018). Three types of mesoporous carbons were tested and compared for PhGs extraction and purification: ordered mesoporous carbon (CMK-3), disordered mesoporous carbon (DMC), and three-dimensional cubic mesoporous carbon (CMK-8). The results indicated that CMK-3 showed the highest adsorption capacity due to its highly specific surface area, its large number of pores and its oxygen-containing functional groups. Finally, Zhao et al. also presented the purification of the acteoside (main PhG from *Cistanche tubulosa*) in its extracts by means of other adsorbing substances such as molecularly imprinted polymers (MIPs) (Lulinski et al., 2016). In their study, the MIPs were designed and synthesized to selectively adsorb acteoside. The effects of different functional monomers, crosslinkers, and MIP solvents were investigated. The MIPs were studied in static adsorption, dynamic adsorption, and selectivity experiments. The results proved that MIPs can be employed as an adsorption material to purify the active ingredients that can be found in plant extracts.

In this Research Topic, we have tried to present the state of the art on the use of novel techniques for the extraction and/or purification of different natural compounds of interest for human health that can be found in a variety of plant matrices. A number of advanced techniques, new materials, and the use of green methodologies have been described and their promising results lead us to think that they will soon be replacing older, traditional methods that demand larger amounts of energy, time, and solvents, with the subsequent environmental and economic negative effects.

## Author Contributions

GB, AA, MF-G, and MR contributed in handling the manuscripts as Guest/Associate guest editors and have also written the editorial. All authors contributed to the article and approved the submitted version.

## Conflict of Interest

The authors declare that the research was conducted in the absence of any commercial or financial relationships that could be construed as a potential conflict of interest.

## References

[B1] Barba F. J. Zhu Z. Koubaa M. Sant'Ana A. S. Orlien V. (2016). Green alternative methods for the extraction of antioxidant bioactive compounds from winery wastes and by-products:a review. Trends Food Sci. Technol. 49, 96–109. 10.1016/j.tifs.2016.01.006

[B2] Chadni Z. Rahaman M. H. Jerin I. Hoque K. Reza M. A. (2017). Extraction and optimisation of red pigment production as secondary metabolites from *Talaromyces verruculosus* and its potential use in textile industries. Mycology 8, 48–57. 10.1080/21501203.2017.1302013

[B3] Feo M. L. Eljarrat E. Barceló D. (2010). Determination of pyrethroid insecticides in environmental samples. TrAC-Trend. Anal. Chem. 29, 692–705. 10.1016/j.trac.2010.03.011

[B4] Lulinski P. Bamburowicz-Klimkowska M. Dana M. Szutowski M. Maciejewska D. (2016). Efficient strategy for the selective determination of dopamine in human urine by molecularly imprinted solid-phase extraction. J. Separ. Sci. 39, 895–903. 10.1002/jssc.201501159 26732188

[B5] Qin G. Ma J. Wei W. Li J. Yue F. (2018). The enrichment of chlorogenic acid from eucommia ulmoides leaves extract by mesoporous carbons. J. Chromatogr. B 1087–1088, 6–13. 10.1016/j.jchromb.2018.04.036 29702354

[B6] Vasireddy S. Morreale B. Cugini A. Song C. Spivey J. J. (2011). Clean liquid fuels from direct coal liquefaction: chemistry, catalysis, technological status and challenges. Energy Environ. Sci. 4, 311–345. 10.1039/C0EE00097C

